# Identification of type III secretion substrates of *Chlamydia trachomatis* using *Yersinia enterocolitica* as a heterologous system

**DOI:** 10.1186/1471-2180-14-40

**Published:** 2014-02-17

**Authors:** Maria da Cunha, Catarina Milho, Filipe Almeida, Sara V Pais, Vítor Borges, Rui Maurício, Maria José Borrego, João Paulo Gomes, Luís Jaime Mota

**Affiliations:** 1 Infection Biology Laboratory, Instituto de Tecnologia Química e Biológica António Xavier, Universidade Nova de Lisboa, Oeiras, Portugal; 2 Department of Infectious Diseases, National Institute of Health, Lisbon, Portugal; 3 Centro de Recursos Microbiológicos (CREM), Departamento de Ciências da Vida, Faculdade de Ciências e Tecnologia, Universidade Nova de Lisboa, Caparica, Portugal; 4 Present address: Centre of Biological Engineering, University of Minho, Campus de Gualtar, Braga, Portugal; 5 Present address: Cambridge Institute for Medical Research, Addenbrooke’s Hospital, Hills Road, Cambridge, United Kingdom

**Keywords:** Bacterial pathogenesis, *Chlamydia*, *Yersinia*, Type III secretion, Effectors

## Abstract

**Background:**

*Chlamydia trachomatis* is an obligate intracellular human pathogen causing ocular and urogenital infections that are a significant clinical and public health concern. This bacterium uses a type III secretion (T3S) system to manipulate host cells, through the delivery of effector proteins into their cytosol, membranes, and nucleus. In this work, we aimed to find previously unidentified *C. trachomatis* T3S substrates.

**Results:**

We first analyzed the genome of *C. trachomatis* L2/434 strain for genes encoding mostly uncharacterized proteins that did not appear to possess a signal of the general secretory pathway and which had not been previously experimentally shown to be T3S substrates. We selected several genes with these characteristics and analyzed T3S of the encoding proteins using *Yersinia enterocolitica* as a heterologous system. We identified 23 *C. trachomatis* proteins whose first 20 amino acids were sufficient to drive T3S of the mature form of β-lactamase TEM-1 by *Y. enterocolitica*. We found that 10 of these 23 proteins were also type III secreted in their full-length versions by *Y. enterocolitica*, providing additional support that they are T3S substrates. Seven of these 10 likely T3S substrates of *C. trachomatis* were delivered by *Y. enterocolitica* into host cells, further suggesting that they could be effectors. Finally, real-time quantitative PCR analysis of expression of genes encoding the 10 likely T3S substrates of *C. trachomatis* showed that 9 of them were clearly expressed during infection of host cells.

**Conclusions:**

Using *Y. enterocolitica* as a heterologous system, we identified 10 likely T3S substrates of *C. trachomatis* (CT053, CT105, CT142, CT143, CT144, CT161, CT338, CT429, CT656, and CT849) and could detect translocation into host cells of CT053, CT105, CT142, CT143, CT161, CT338, and CT429. Therefore, we revealed several *C. trachomatis* proteins that could be effectors subverting host cell processes.

## Background

*Chlamydiae* are a large group of obligate intracellular bacteria that includes human pathogens (e.g. *Chlamydia trachomatis* or *C. pneumoniae*), animal pathogens (e.g. *C. abortus*, *C. caviae*, *C. felis*, or *C. muridarum*), or symbionts of free-living amoebae. Among *Chlamydiae*, *C. trachomatis* is a particular clinical and public health concern, being the leading cause of infectious blindness in developing countries [1] and the most prevalent sexually transmitted bacteria worldwide [2].

Like all *Chlamydiae*, *C. trachomatis* undergoes a developmental cycle involving the inter-conversion between two morphologically distinct forms: a non-replicative infectious form, the elementary body (EB), and a replicative non-infectious form, the reticulate body (RB) [3]. Throughout its developmental cycle, *C. trachomatis* uses a type III secretion system (T3SS) to translocate several effector proteins across the host cell plasma membrane and the inclusion membrane [4,5]. These T3S effectors are thought to play a central role in bacterial invasion [6,7] and exit of host cells [8], and in the subversion of various host cell processes [9-16]. There are, however, chlamydial effectors, such as CPAF/CT858 or CT441, which are not T3S substrates [4].

Given their likely central role during infection, considerable efforts have been placed at identifying chlamydial effectors. This is not a trivial task because the amino acid sequence of most effectors does not display significant similarity to proteins of known function. Additionally, T3S substrates, which should comprise the bulk of *Chlamydia* effectors, contain no easily recognizable secretion signal. Moreover, in spite of the recent development of systems for transformation of *Chlamydia*[17,18], for a long time no methods have been available for genetic manipulation of these bacteria. To overcome these obstacles, chlamydial effectors have been searched: i) by systematic phenotypic analyses of yeast *Saccharomyces cerevisiae* expressing individual chlamydial proteins [19]; ii) by using *Salmonella*[20], *Shigella*[15,21-23], or *Yersinia*[13,14,24-27] as genetically tractable heterologous host bacteria carrying well characterized T3SSs; or iii) by complex computational predictions of T3S signals [28-30]. The subsequent use of specific antibodies enabled to detect translocation into host cells of some of the *C. trachomatis* proteins singled out in these searches, such as in the case of Tarp/CT456 [25], CT694 [14], CopN/CT089 [24], Cap1/CT529 [31], CT620 [22], CT621 [22,32], CT711 [22], lipid-droplet associated (Lda) proteins Lda1/CT156, Lda2/CT163, and Lda3/CT473 [33], Nue/CT737 [15], or of a group of proteins containing a hydrophobic motif thought to mediate their insertion into the inclusion membrane (Inc proteins) [12,34]. Moreover, the direct use of antibodies raised against particular *C. trachomatis* proteins (CT311, CT622, CT795, GlgA/CT798, HtrA/CT823, or Pgp3) revealed their presence in the host cell cytosol or nucleus of infected cells [35-40]. Finally, the *in vitro* deubiquitinase activity of *Chla*DUB1/CT868 and of *Chla*DUB2/CT867 [41], and the capacity of *Chla*DUB1/CT868 to suppress the NF-κB pathway in transfected cells [42], indicate that these two proteins should be effectors.

In this work, we have surveyed the genome of *C. trachomatis* mostly for genes encoding uncharacterized proteins that were not described before as T3S substrates. We then used *Yersinia enterocolitica* as a heterologous system to identify 10 novel likely T3S substrates of *C. trachomatis* and real-time quantitative PCR (RT-qPCR) to show that 9 of the genes encoding these proteins are clearly expressed during the bacterial developmental cycle. Furthermore, we showed that 7 of the 10 likely T3S substrates of *C. trachomatis* could be translocated into host cells by *Y. enterocolitica*. Therefore, we identified several novel putative effectors of *C. trachomatis*.

## Methods

### Cell culture, bacterial strains and growth conditions

HeLa 229 (ATCC) cells were maintained in Dulbecco’s modified Eagle’s medium (DMEM; Invitrogen) supplemented with 10% (v/v) foetal bovine serum (FBS; Invitrogen) at 37°C in a humidified atmosphere of 5% (v/v) CO_2_. *C. trachomatis* L2/434/Bu (from ATCC) was propagated in HeLa 229 cells using standard techniques [43]. *Escherichia coli* TOP10 (Invitrogen) was used for construction and purification of the plasmids. *Yersinia enterocolitica* ΔHOPEMT (MRS40 pIML421 [*yopH*_Δ1-352_, *yopO*_Δ65-558_, *yopP*_*23*_, *yopE*_*21*_, *yopM*_*23*_, *yopT*_*135*_]), deficient for the *Yersinia* T3S effectors YopH, O, P, E, M, and T, but T3S-proficient [44] and T3S-deficient *Y. enterocolitica* ΔHOPEMT ΔYscU (MRS40 pFA1001 [*yopH*_Δ1-352_, *yopO*_Δ65-558_, *yopP*_*23*_, *yopE*_*21*_, *yopM*_*23*_, *yopT*_*135*_, *yscU*_*Δ1-354*_) [45] were used for T3S assays. The *yscU* gene encodes an essential component of the *Y. enterocolitica* T3S system, and the *yscU*_*Δ1-354*_ mutation is non-polar [46]. *E. coli* or *Y. enterocolitica* were routinely grown in liquid or solid Luria-Bertani (LB) medium (NZYtech) with the appropriate antibiotics and supplements. Plasmids were introduced into *E. coli* or *Y. enterocolitica* by electroporation.

### DNA manipulations, plasmids, and primers

The plasmids used in this work and their main characteristics are detailed in Additional file 1: Table S1. The DNA primers used in their construction are shown in Additional file 2: Table S2. Plasmids were constructed and purified with proof-reading Phusion DNA polymerase (Finnzymes), restriction enzymes (MBI Fermentas), T4 DNA Ligase (Invitrogen), DreamTaq DNA polymerase (MBI Fermentas), DNA clean & concentrator™-5 Kit and Zymoclean™ Gel DNA Recovery kit (Zymo Research), and purified with GeneElute Plasmid Miniprep kit (Sigma), according to the instructions of the manufacturers. In brief, to analyze T3S signals we constructed plasmids harboring hybrid genes encoding the first 10, 15, 20, or 40 amino acids of each protein *(C. trachomatis* proteins, SycT and YopE) and the mature form of TEM-1 β-lactamase (TEM-1) [47]. These hybrids were made using as vector pLJM3, a low-copy plasmid which enables expression of the cloned genes driven by the promoter of the *Y. enterocolitica yopE* gene [48], either by overlapping PCR or by using a cloning strategy previously described for the construction of plasmids encoding Inc-TEM-1 hybrid proteins [45]. To analyze secretion of full-length *C. trachomatis* proteins, we constructed plasmids expressing the proteins C-terminally tagged with a haemagglutinin (HA) epitope. For this, the genes were amplified by PCR from chromosomal DNA of strain L2/434/Bu using a reverse primer with a sequence complementary to the transcribed strand of the DNA encoding the HA-epitope. PCR products digested with the appropriate enzymes were ligated into pLJM3 [48]. The accuracy of the nucleotide sequence of all the inserts in the constructed plasmids was checked by DNA sequencing.

### *Y. enterocolitica* T3S assays

T3S assays were done as previously described [46]. We used *Y. enterocolitica* ΔHOPEMT or ΔHOPEMT ΔYscU strains carrying the plasmids described in Additional file 1: Table S1. The proteins in bacterial pellets and culture supernatants were analyzed by immunoblotting, and the amounts of protein in bacterial pellets and/or culture supernatants were estimated from images of immunoblots with Image Lab (Bio-Rad). Where appropriate, we calculated the percentage of secretion as the ratio between the amounts of secreted protein (in the culture supernatant fraction) relative to the total amount of protein (in the culture supernatant and in the bacterial pellet fractions). The results from the quantifications are the average ± standard error of the mean (SEM) from at least three independent experiments. Detailed results for each protein analyzed are in Additional file 3: Table S3.

### *Y. enterocolitica* translocation assays

Analyses of protein translocation into host cells by *Y. enterocolitica* were done essentially as previously described [49,50]. In brief, *Y. enterocolitica* strains were grown in brain heart infusion (BHI; Scharlau) medium overnight at 26°C with continuous shaking (130 rpm). Bacteria were then diluted to an optical density at 600 nm of 0.2 in fresh BHI and cultured in the same conditions for 2 h. Subsequently, the *yop* regulon was induced by incubation for 30 min in a shaking water bath (130 rpm) at 37°C. Bacteria were then washed with DMEM supplemented with 10% (v/v) FBS and added to HeLa 229 cells, grown overnight in 24-well plates (1x10^5^ cells/well), by using a multiplicity of infection of 50. The infected cells were incubated at 37°C in a humidified atmosphere of 5% (v/v) CO_2_. After 3 h of incubation, extracellular bacteria were killed by adding gentamicin (50 μg/ml), and the cells were incubated in the same conditions for additional 2 h. The infected cells were then harvested on ice, washed with phosphate-buffered saline (PBS), ressuspended in PBS containing 0.1% (v/v) Triton X-100 and a protease inhibitor cocktail (Sigma), and incubated for 10 min on ice. The samples were centrifuged (15,000 *g* for 15 min at 4°C) and Triton-soluble and Triton-insoluble HeLa cell lysates were loaded on sodium dodecyl sulfate-12% (v/v) polyacrilamide gels. After electrophoresis, the gels were processed for immunoblotting using 0.2 μm pore-size nitrocellulose membranes (BioRad).

### Immunoblotting

The following antibodies were used for immunoblotting: rat monoclonal anti-HA (clone 3F10; Roche; used at 1:1000), mouse monoclonal anti-TEM-1 (QED Bioscience; 1:500), rabbit polyclonal anti-SycO (1:1000) [51], and mouse monoclonal anti-tubulin (clone B-5-1-2; Sigma; 1:1000). Immunoblot detection was done with horseradish peroxidase-conjugated secondary antibodies (GE Healthcare and Jackson ImmunoResearch), Western Lightning *Plus*-ECL (Perkin Elmer), and a ChemiDoc XRS + system (BioRad) or exposure to Amersham Hyperfilm ECL (GE Healthcare). All quantitative analyses were done with immunoblot images obtained using ChemiDoc XRS + (BioRad).

### Real-time quantitative PCR

The expression of the newly identified candidate T3S substrates during the developmental cycle of *C. trachomatis* L2/434 was estimated by determining mRNA levels at different times post-infection by real-time quantitative PCR (RT-qPCR). These experiments were done as previously described [45]. Primers (available upon request) were designed using Primer Express (Applied Biosystems). The RT-qPCR assays were done using the ABI 7000 SDS, SYBR green chemistry, and optical plates (Applied Biosystems), as previously described [52]. At each time point, raw RT-qPCR data for each gene were normalized against the data obtained for the 16S rRNA transcript, as it was previously demonstrated that this is an adequate endogenous control [52]. The final results were based on three independent experiments.

## Results

### Selection of *C. trachomatis* proteins analyzed in this work

To search for previously unidentified T3S substrates of *C. trachomatis*, we first surveyed the genome of strain L2/434 for genes encoding mostly uncharacterized proteins, or with a putative biochemical activity compatible with the function of a T3S effector (e.g., proteases). Among these genes, we selected those encoding proteins not predicted to have a signal sequence characteristic of the general secretory pathway (according to Psortb v3.0) and that had not been previously analyzed experimentally for the presence of a T3S signal. This singled out 32 proteins (CT016, CT017, CT031, CT051, CT053, CT080, CT105, CT142, CT143, CT144, CT153, CT161, CT172, CT273, CT277, CT289, CT309, CT330, CT338, CT386, CT425, CT568, CT583, CT590, CT631, CT635, CT656, CT696, CT702, CT837, CT845, and CT849; we used the nomenclature of the annotated *C. trachomatis* D/UW3 strain [53]; the names of the corresponding genes as annotated for strain L2/434 [54] can be found in Additional file 3: Table S3). Furthermore, for comparison purposes, we considered proteins that had been tested for the presence of a T3S signal using *Shigella flexneri* as a heterologous bacteria: eight proteins whose first ~40 amino acids of the corresponding *C. pneumoniae* homologs did not drive secretion of an adenylate cyclase (Cya) reporter protein by *S. flexneri* (CT066, CT429, GrgA/CT504, CT538, CT584, CT768, CT779, CT814), and three proteins whose N-terminal region of the *C. pneumoniae* homologs drove secretion of a Cya reporter protein by *S. flexneri* (CT203, CT577, CT863) [21]. Please note that at the time this work was initiated GrgA/CT504 was an uncharacterized protein; however, it was recently described as a transcriptional activator [55]. Finally, throughout this study we used as positive controls a *C. trachomatis bona-fide* T3S effector (CT694) [14] and a *C. trachomatis* likely T3S substrate (CT082) that we had previously identified [26], and which was recently independently confirmed [27], and as negative control a predicted ribosomal protein (RplJ/CT317).

In summary, in experiments that will be described below, we analyzed T3S signals in 46 *C. trachomatis* proteins (~5% of all proteins encoded by the L2/434 strain): 32 hypothetical proteins previously not analyzed experimentally for T3S signals, 11 proteins whose *C. pneumoniae* homologs were previously analyzed for T3S signals using *S. flexneri* as heterologous system, and 3 controls. In the selection of these proteins, we did not consider predictions made by any of the published *in silico* methods that suggest putative T3S substrates [28-30,56].

### The first 20 amino acids of *C. trachomatis* T3S substrates are sufficient to drive efficient secretion of TEM-1 hybrid proteins by *Y. enterocolitica*

**Figure 1:**
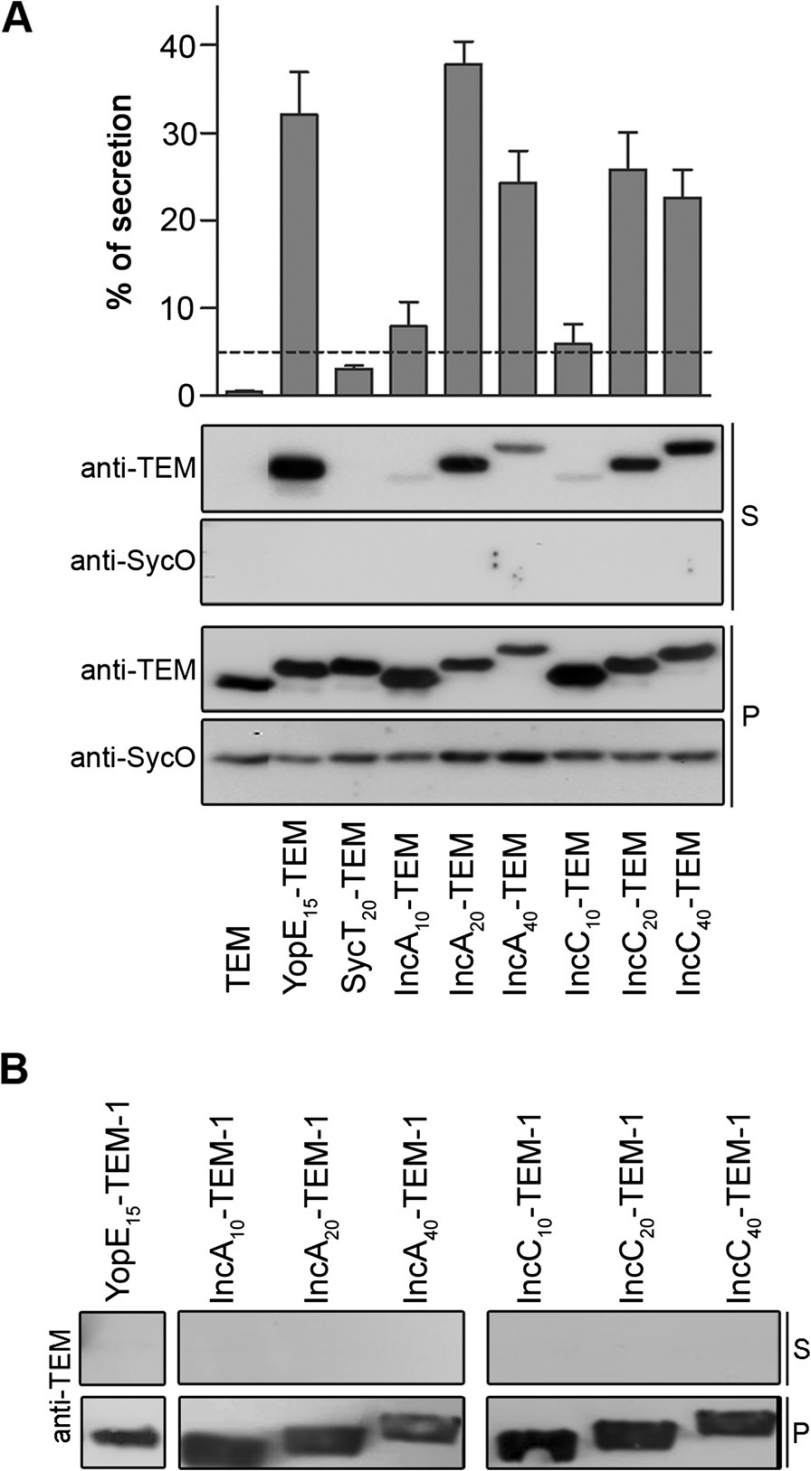
**The first 20 amino acids of known *C. trachomatis *T3S substrates (IncA or IncC) are sufficient to efficiently drive T3S of TEM-1 hybrid proteins by *Y. enterocolitica*. ***Y. enterocolitica* T3S-proficient (ΔHOPEMT) **(A)** and T3S-defective (ΔHOPEMT ΔYscU) **(B)** were used to analyze secretion of hybrid proteins comprising the first 10, 20, or 40 amino acids of *C. trachomatis* IncA or IncC, or the first 15 or 20 amino acids of *Y. enterocolitica* YopE or SycT, respectively, fused to the mature form of TEM-1 β-lactamase (TEM-1). Immunoblots show the result of T3S assays in which proteins in culture supernatants (S, secreted proteins) and in bacterial pellets (P, nonsecreted proteins) from ~5x10^7^ bacteria were loaded per lane. The first 15 amino acids of the *Yersinia* effector YopE correspond to an archetypal T3S signal [57,58], and YopE_15_-TEM-1 was used as positive control; SycT and SycO are strictly cytosolic *Yersinia* T3S chaperones [44,51]. SycT_20_-TEM-1 was a negative control for the T3S assays. Immunodetection of SycO ensured that the presence of TEM-1 hybrid proteins in the culture supernatants was not a result of bacterial lysis or contamination. The percentage (%) of secretion of each TEM-1 hybrid was calculated by densitometry, as the ratio between the amount of secreted and total protein. The threshold to decide whether a protein was secreted was set to 5% (dashed line), based on the % of secretion of SycT_20_-TEM-1. Data are the mean ± SEM from at least 3 independent experiments.

We previously used TEM-1 as a reporter protein to analyze T3S signals in *C. trachomatis* Inc proteins, using *Y. enterocolitica* as a heterologous system [45]. However, before analyzing T3S signals in the proteins that we selected to study in this work (see above), we sought to ascertain the optimal amino acid length of the chlamydial T3S signal that drives secretion of TEM-1 hybrid proteins in *Yersinia*. For this, we analyzed secretion of hybrid proteins comprising the first 10, 20 and 40 amino acids of known *C. trachomatis* T3S substrates (IncA or IncC) fused to TEM-1 (IncA_10_-TEM-1, IncA_20_-TEM-1, IncA_40_-TEM-1, IncC_10_-TEM-1, IncC_20_-TEM-1, IncC_40_-TEM-1) by T3S-proficient (ΔHOPEMT) or T3S-deficient (ΔHOPEMT ΔYscU) *Y. enterocolitica* (Figure 1). As negative controls we analyzed secretion by *Y. enterocolitica* ΔHOPEMT of TEM-1 alone and of a hybrid protein comprising the first 20 amino acids of the *Yersinia* T3S chaperone SycT to TEM-1 (SycT_20_-TEM-1), and as positive control we analyzed secretion by ΔHOPEMT of a fusion of the first 15 amino acids of the *Yersinia* effector YopE to TEM-1 (YopE_15_-TEM-1) (Figure 1), an archetypal T3S signal [57,58]. Bacteria expressing these proteins were incubated under T3S-inducing conditions, as described in Methods. As expected, and in agreement to what we previously reported [45], mature TEM-1 alone was not secreted and the SycT_20_-TEM-1 fusion showed a percentage of secretion of 3.0 (SEM, 0.3). Based on this, to decide if a TEM-1 hybrid was secreted or not, we set the threshold of percentage of secretion to 5 (Figure 1A). The six Inc-TEM-1 hybrid proteins were type III secreted (Figure 1A and B). However, IncA_10_-TEM-1 and IncC_10_-TEM-1 were secreted less efficiently than YopE_15_-TEM-1, while IncA_20_-TEM-1, IncA_40_-TEM-1, IncC_20_-TEM-1 and IncC_40_-TEM-1 were secreted at levels comparable to YopE_15_-TEM-1 (Figure 1A). Overall, these experiments indicated that the first 20 amino acids of *C. trachomatis* T3S substrates are sufficient to drive secretion of TEM-1 hybrid proteins by *Y. enterocolitica* ΔHOPEMT as efficiently as the first 15 amino acids of the *Yersinia* effector YopE.

### Identification of T3S signals in *C. trachomatis* proteins

**Figure 2:**
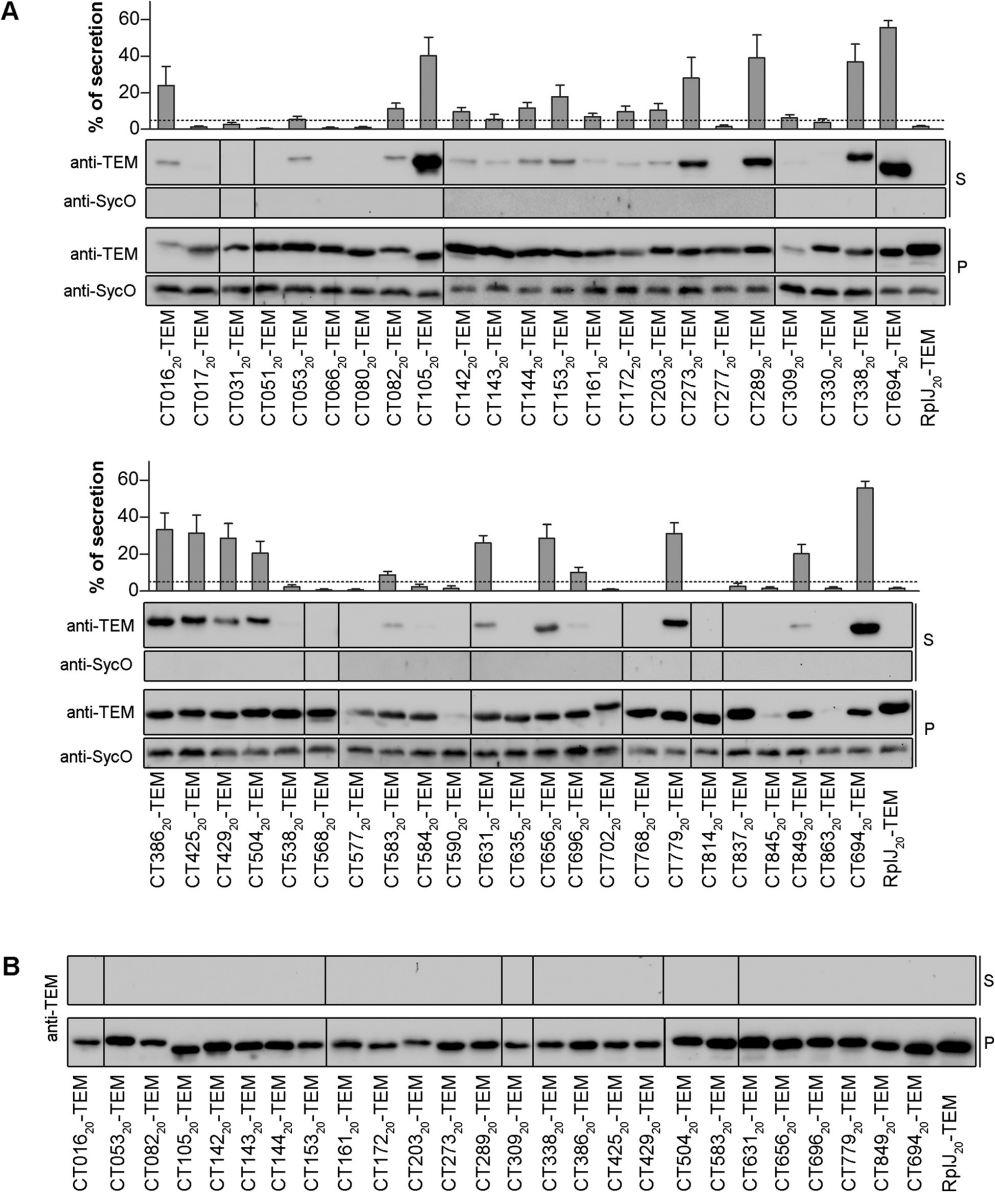
**Identification of T3S signals in *C. trachomatis *proteins using *Y. enterocolitica *as a heterologous system. ***Y. enterocolitica* T3S-proficient (ΔHOPEMT) **(A)** and T3S-defective (ΔHOPEMT ΔYscU) **(B)** were used to analyze secretion of hybrid proteins comprising the first 20 amino acids of selected *C. trachomatis* proteins or the first 20 amino acids of *Y. enterocolitica* SycT fused to the mature form of TEM-1 β-lactamase (TEM-1). Immunoblots show the result of T3S assays in which proteins in culture supernatants (S, secreted proteins) and in bacterial pellets (P, non-secreted proteins) from ~2.5x10^8^ and ~5x10^7^ bacteria, respectively, were loaded per lane. TEM-1 hybrids of the known *C. trachomatis* T3S substrates CT082 [26,27] and CT694 [14] were used as positive controls. SycT and SycO are strictly cytosolic *Yersinia* T3S chaperones [44,51]. SycT_20_-TEM-1 was a negative control for the T3S assays. Immunodetection of SycO ensured that the presence of TEM-1 hybrid proteins in the culture supernatants was not a result of bacterial lysis or contamination. The percentage (%) of secretion of each TEM-1 hybrid was calculated by densitometry, as the ratio between the amount of secreted and total protein. The threshold to decide whether a protein was secreted was set to 5% (dashed line), based on the% of secretion of SycT_20_-TEM-1. Data are the mean ± SEM from at least 3 independent experiments.

To identify T3S signals in the selected 46 *C. trachomatis* proteins, we analyzed secretion of fusions to TEM-1 of the first 20 amino acids of each of these proteins by T3S-proficient *Y. enterocolitica* ΔHOPEMT. These experiments revealed 24 *C. trachomatis* proteins whose first 20 amino acids drove secretion of TEM-1 hybrid proteins by *Y. enterocolitica* (Figure 2A). Owing to lack of expression, or very low expression levels, it was not possible to conclude if the TEM-1 hybrids comprising the N-terminal region of CT590, CT845 and CT863 were secreted (Figure 2A). By individually introducing the plasmids encoding the TEM-1 hybrid proteins that were secreted into T3S-deficient *Y. enterocolitica* ΔHOPEMT ΔYscU and performing T3S assays, we confirmed that secretion of the proteins was dependent on a functional T3SS (Figure 2B). The percentage of secretion of the different hybrid proteins that were secreted varied considerable, between 56% (SEM, 4) for CT694_20_-TEM-1 to 5% (SEM, 2) for CT143_20_-TEM-1 (Figure 2B). Overall, this confirmed a T3S signal in CT203, which has been previously shown to be a T3S substrate [21], and revealed T3S signals in 23 previously T3S substrates of *C. trachomatis*.

### Analysis of the secretion of the newly identified candidate T3S substrates of *C. trachomatis* as full-length proteins

We next analyzed if the 23 *C. trachomatis* proteins carrying newly identified T3S signals, and also CT203 and the controls (CT082, CT694 and RplJ), were secreted as full-length proteins by *Y. enterocolitica* ΔHOPEMT. The rationale for these experiments was that some proteins cannot be type III secreted even with a T3S signal grafted at their N-termini [59-62], possibly because the secretion channel is too narrow (inner diameter of 2–3 nm [63]) to accommodate tightly folded proteins. For example, while we showed that YopE_15_-TEM-1 is efficiently type III secreted, hybrid proteins containing the first 15 or 16 amino acids of YopE fused to mouse dihydrofolate reductase (DHFR) are not type III secreted by *Y. enterocolitica*[59,60]. This indicates that most T3S substrates must have particular folding properties that are compatible with them being type III secreted proteins. Based on this, we predicted that if the full-length version of chlamydial proteins were type III secreted by *Yersinia* this would be an additional indication that they can be T3S substrates. However, lack of secretion of the full-length proteins would not preclude that they could be T3S substrates, as they may require *Chlamydia*-specific chaperones, not present in *Yersinia*[64].

**Figure 3:**
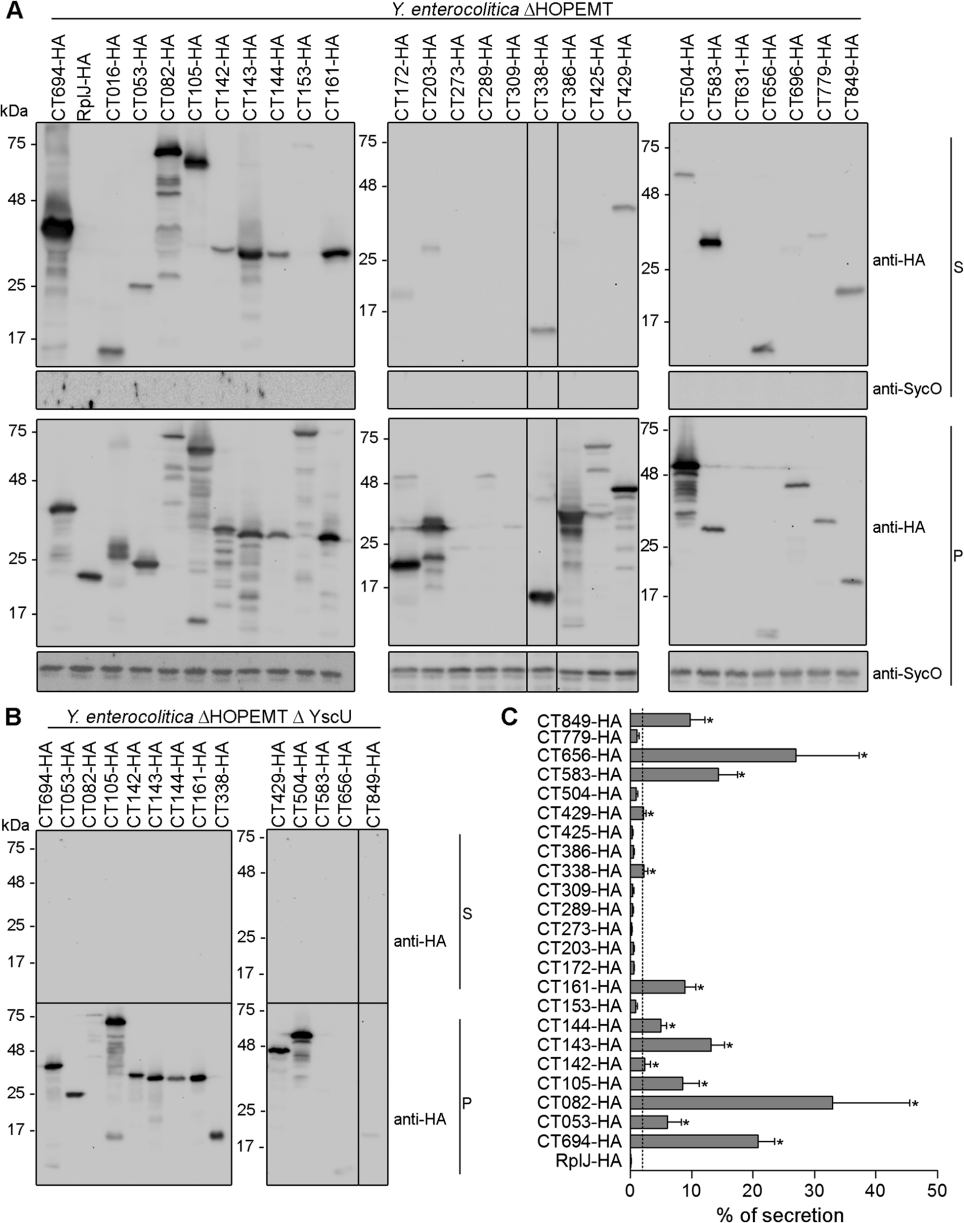
**Analysis of the T3S of *C. trachomatis *full-length proteins by *Y. enterocolitica*. ***Y. enterocolitica* T3S-proficient (ΔHOPEMT) **(A)** and T3S-defective (ΔHOPEMT ΔYscU) **(B)** were used to analyze secretion of full-length *C. trachomatis* proteins with a C-terminal HA epitope tag. Immunoblots show the result of T3S assays in which proteins in culture supernatants (S, secreted proteins) and in bacterial pellets (P, non-secreted proteins) from ~5 x 10^8^ and ~5 x 10^7^ bacteria, respectively, were loaded per lane. The known *C. trachomatis* T3S substrates CT082 [26,27] and CT694 [14] were used as positive controls, and the *C. trachomatis* ribosomal protein RplJ was used as a negative control. SycO is a strictly cytosolic *Yersinia* T3S chaperone [44,51] and its immunodetection ensured that the presence of HA-tagged proteins in the culture supernatants was not a result of bacterial lysis or contamination. **(C)** The percentage (%) of secretion of each protein by *Y. enterocolitica* ΔHOPEMT was calculated by densitometry, as the ratio between the amount of secreted and total protein. The threshold to decide whether a protein was secreted was set to 2% (dashed line), based on the % of secretion of RplJ-HA. Data are the mean ± SEM from at least 3 independent experiments.

To analyze secretion of full-length *C. trachomatis* proteins by *Y. enterocolitica* we used plasmids expressing the chlamydial proteins with an HA tag at their C-termini. The plasmids were introduced into *Y. enterocolitica* ΔHOPEMT and T3S assays were performed. In these experiments, the percentage of secretion of the positive controls (CT694-HA and CT082-HA) was between 20-30% and the percentage of secretion of the negative control (RplJ-HA) was 0.13% (SEM, 0.05). Based on these results, in experiments involving full-length proteins of newly identified chlamydial T3S substrates we set a conservative threshold of 2% to decide whether a protein was secreted or not. This defined a group of 11 proteins that in their full-length version were secreted by *Y. enterocolitica* ΔHOPEMT: CT053-HA, CT105-HA, CT142-HA, CT143-HA, CT144-HA, CT161-HA, CT338-HA, CT429-HA, CT583-HA, CT656-HA, and CT849-HA (Figure 3A and B). To test if secretion of these proteins was dependent on a functional T3SS, the plasmids carrying their encoding genes, as well as plasmids encoding positive controls CT694-HA or CT082-HA, were individually introduced into T3S-deficient *Y. enterocolitica* ΔHOPEMT ΔYscU. With the exception of CT583-HA, which for unknown reasons was very poorly expressed by *Y. enterocolitica* ΔHOPEMT ΔYscU, these assays indicated that the other 10 proteins analyzed were type III secreted (Figure 3C).

Secretion of full-length CT153-HA, CT172-HA, CT203-HA, CT386-HA or CT425-HA by *Y. enterocolitica* could occasionally be seen by immunoblotting (Figure 3A); however, this was not always reproducible and individual average percentage of secretion of these proteins was in all cases below 2% (Figure 3B). We did not detect significant amounts of CT273-HA, CT289-HA, CT309-HA, or CT631-HA in culture supernatants (Figure 3A and Additional file 3: Table S3), but as their levels of expression were either extremely low (CT273-HA, CT289-HA, and CT309-HA) or undetectable (CT631-HA) it was not possible to draw conclusions about secretion of these proteins. Furthermore, CT016-HA, and possibly CT696-HA (barely visible in Figure 3A), were immunodetected in the culture supernatant fraction in a form that migrated on SDS-PAGE at a molecular weight much lower than the one predicted from their amino acid sequence (27 kDa and 46 kDa, respectively), while in the bacterial pellet fraction their migration on SDS-PAGE corresponded roughly to their predicted molecular weight (Figure 3A). This suggests that the proteins could be cleaved during secretion, unstable in the culture supernatant, or their encoding genes possess internal Shine-Dalgarno sequences. Regardless of the exact reason, we could not confidently analyze whether CT016-HA and CT696-HA were secreted or not.

Overall, the full set of T3S assays revealed 10 proteins (CT053, CT105, CT142, CT143, CT144, CT161, CT338, CT429, CT656, and CT849) as newly identified likely T3S substrates of *C. trachomatis*, and therefore as possible effectors.

### CT053, CT105, CT142, CT143, CT161, CT338, and CT429 can be translocated into host cells by *Y. enterocolitica*

**Figure 4:**
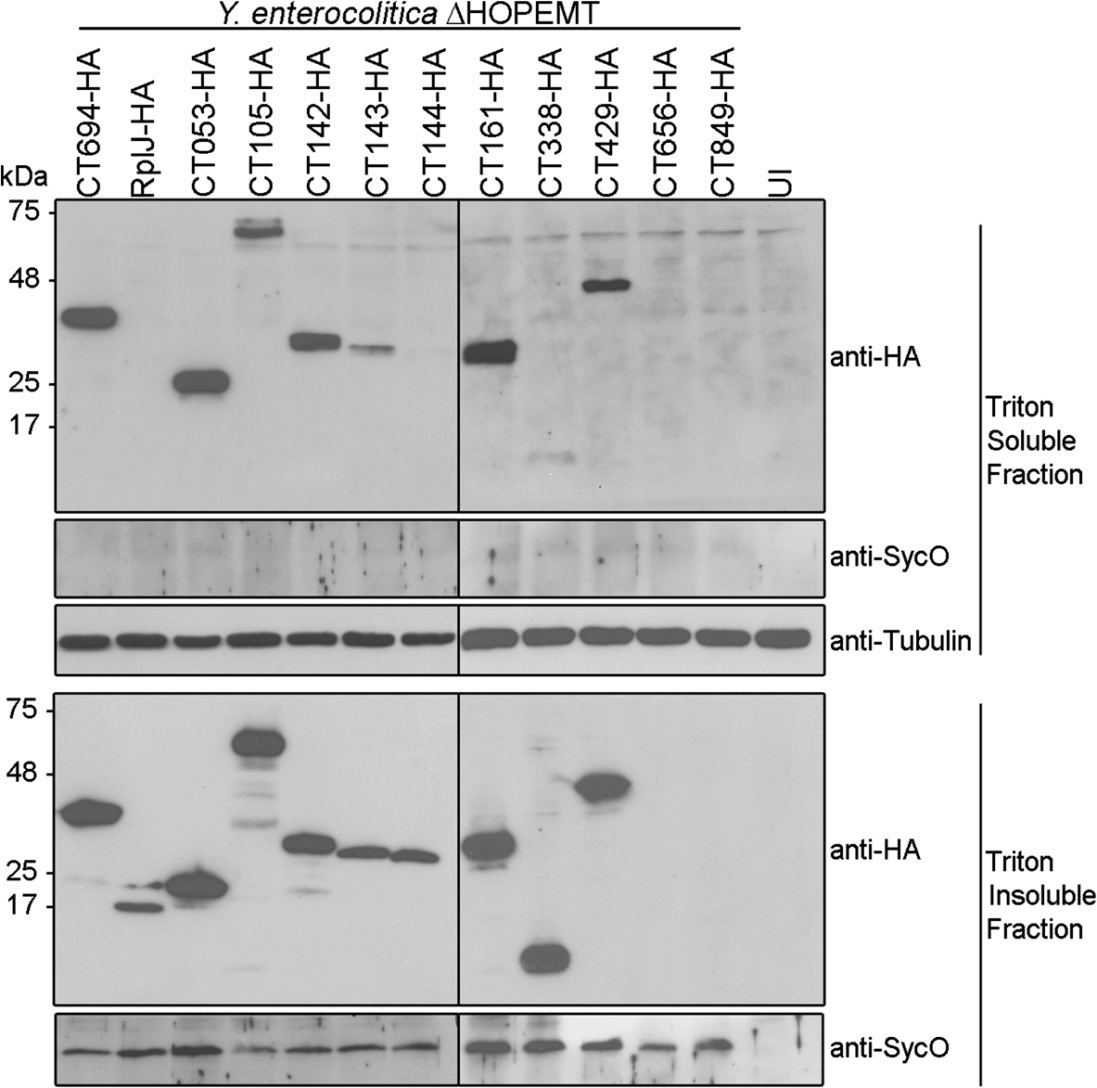
**Translocation of *C. trachomatis *proteins into the cytoplasm of HeLa cells by *Y. enterocolitica*.** HeLa cells were left uninfected (UI) or infected with *Y. enterocolitica* ΔHOPEMT strains expressing the indicated HA-tagged proteins. After 3 h of infection, extracellular bacteria were killed by the addition of gentamicin and the infected cells were incubated for additional 2 h. The infected cells were then fractionated into Triton-soluble and Triton-insoluble cell lysates that were subsequently analyzed by immunoblotting using anti-HA, anti-SycO and anti-tubulin antibodies, as indicated. Presence of HA-tagged proteins in the Triton-soluble cell lysates is indicative of translocation into the cytoplasm of HeLa cells. SycO is a strictly cytosolic *Yersinia* T3S chaperone [44,51] and its immunodetection ensured that the presence of HA-tagged proteins in the Triton-soluble cell lysates was not a result of bacterial lysis during the fractionation. Additionally, the incapacity to detect HA-tagged RplJ (a *C. trachomatis* ribosomal protein) in the Triton-soluble cell lysates further indicated that this fraction did not contain bacteria or non-translocated bacterial proteins. Tubulin served as a loading control of the Triton-soluble cell lysates. The images shown are representative of three independent experiments.

We next analyzed if the newly identified likely T3S substrates of *C. trachomatis* had the capacity of being translocated into host cells, by using *Y. enterocolitica* as a heterologous system. For this, *Y. enterocolitica* ΔHOPEMT harboring plasmids encoding C-terminal HA-tagged newly identified likely T3S substrates of *C. trachomatis* (CT053-HA, CT105-HA, CT142-HA, CT143-HA, CT144-HA, CT161-HA, CT338-HA, CT429-HA, CT656-HA, or CT849-HA), a positive control (CT694-HA) or a negative control (RplJ-HA), were used to infect human epithelial HeLa cells. We then used Triton X-100 fractionation of the infected cells followed by immunoblotting analysis of Triton-soluble and insoluble HeLa cell lysates to monitor protein translocation into host cells. As expected, we found CT694-HA in the Triton-soluble fraction, which showed that this protein was delivered into the cytoplasm of HeLa cells, and only detected RplJ-HA in the Triton-insoluble fraction (Figure 4), which confirmed that this protein remained within the bacteria (and that the fractionation procedure did not lyse the bacteria). Among the 10 likely T3S substrates of *C. trachomatis* under analysis, we could not detect CT656-HA or CT849-HA in both the Triton-soluble and Triton-insoluble fractions. It is possible that in the experimental conditions used in this study CT656-HA or CT849-HA are translocated in minute and undetectable amounts and/or that they are degraded either after translocation or within the bacteria. Regardless of the exact scenario, these results did not enable us to conclude about the capacity of CT656-HA and CT849-HA of being translocated into host cells. However, we could consistently detect CT053-HA, CT105-HA, CT142-HA, CT143-HA, CT161-HA, CT338-HA and CT429-HA in the Triton-soluble fraction (Figure 4), indicating that these proteins were injected into the cytoplasm of HeLa cells by *Y. enterocolitica*. We could also occasionally detect small amounts of CT144-HA in the Triton-soluble fraction (barely visible in Figure 4).

In summary, these experiments showed that CT053-HA, CT105-HA, CT142-HA, CT143-HA, CT161-HA, CT338-HA and CT429-HA have the capacity of being translocated into infected host cells further suggesting that the endogenous *C. trachomatis* proteins could be effectors. The results do not preclude that CT144, CT656 or CT849 could be effectors, but the evidence is not as strong as for the other 7 proteins.

### Expression of genes encoding newly identified likely T3S substrates during development of *C. trachomatis*

**Figure 5:**
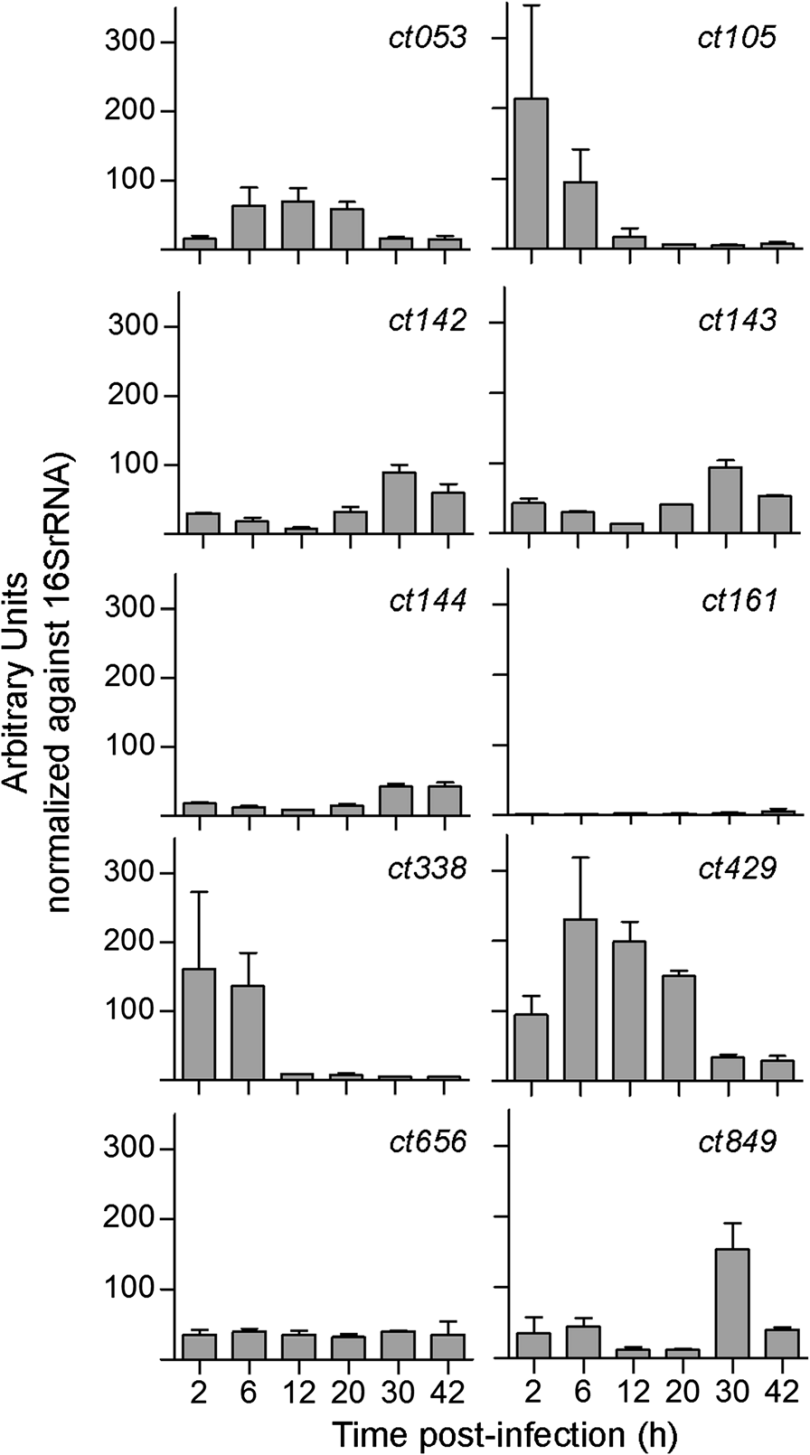
**mRNA levels of newly identified putative effectors during the developmental cycle of *C. trachomatis*.** The mRNA levels of *ct053*, *ct105*, *ct142*, *ct143*, *ct144*, *ct161*, *ct338*, *ct429*, *ct656*, and *ct849* were analyzed by RT-qPCR during the developmental cycle of *C. trachomatis* strain L2/434, at the indicated time-points. The expression values (mean ± SEM) resulted from raw RT-qPCR data (10^5^) of each gene normalized to that of the 16 s rRNA gene and are from three independent experiments.

To test if the newly identified likely T3S substrates, and possible effectors, of *C. trachomatis* (CT053, CT105, CT142, CT143, CT144, CT161, CT338, CT429, CT656, and CT849) were expressed during infection, and to gain insights of when they could be acting during the developmental cycle, we analyzed by RT-qPCR the mRNA levels of their encoding genes during the developmental cycle of strain L2/434, at 2, 6, 12, 20, 30 and 42 h post-infection. While *ct053*, *ct105*, *ct142*, *ct143*, *ct144*, *ct338*, *ct429*, *ct656*, and *ct849* displayed significant mRNA levels in more than one of the time-points analyzed, *ct161* showed only vestigial levels of expression throughout the cycle (Figure 5). The mRNA levels of *ct105* and *ct338* were < 5-fold higher at 2–6 h post-infection than in any other of the time-points analyzed (Figure 5), suggesting that the encoded proteins should function at early-cycle. The mRNA levels of *ct053* and *ct429* were higher between 6 and 20 h post-infection (Figure 5), suggesting that the encoded proteins might act from early to mid cycle. The mRNA levels of *ct142*, *ct143*, *ct144* and *ct849* were higher at the later time points analyzed (30–42 h post-infection). However, while *ct142*, *ct143*, and *ct144* were expressed at similar levels at 30 and 42 h post-infection, *ct849* showed a distinct peak of expression at 30 h post-infection (Figure 5). Therefore, CT142, CT143, CT144 could function either at late or early cycle, and CT849 might probably acts at late cycle. Finally, the mRNA levels of *ct656* were constant at all time-points analyzed (Figure 5), suggesting that CT656 could function throughout the cycle. Regarding *ct161*, when comparing the higher mRNA levels detected for each of the genes analyzed, those of *ct161* were < 6-fold lower than those of any of the other genes tested (Figure 5). Therefore, in the experimental conditions used, CT161 may not be expressed by strain L2/434. In summary, the RT-qPCR experiments supported that CT053, CT105, CT142, CT143, CT338, and CT429, and also CT144, CT656, or CT849, could be *C. trachomatis* T3S effectors, possibly acting at different times of the developmental cycle.

## Discussion

Earlier studies using heterologous systems have led to the identification of several *bona-fide* or putative *C. trachomatis* T3S effectors [13-15,21,22,24-27]. While these and other analyses covered a significant portion of all *C. trachomatis* proteins, we hypothesized that there could be previously unidentified T3S substrates. By combining basic bioinformatics searches, exhaustive T3S assays, translocation assays, and analyses of chlamydial gene expression in infected cells, we revealed 10 *C. trachomatis* proteins (CT053, CT105, CT142, CT143, CT144, CT161, CT338, CT429, CT656, and CT849) as likely T3S substrates and possible effectors. In particular, CT053, CT105, CT142, CT143, CT338, and CT429 were type III secreted by *Y. enterocolitica*, could be translocated into host cells, and their encoding genes were clearly expressed in *C. trachomatis* strain L2/434. Therefore, these 6 proteins have a high likelihood of being effectors. However, additional future studies are required to show that all of these 10 proteins are indeed translocated by *C. trachomatis* into host cells and to show that they are *bona-fide* effectors, i.e., that they interfere with host cell processes.

Among the likely T3S effectors of *C. trachomatis* that we identified, CT105 and CT142 have been previously singled out as possible modulators of host cell functions, based on the phenotypic consequences of their ectopic expression in yeast *S. cerevisiae*[19]. In addition, the genes encoding CT142, CT143, and CT144 have been shown to be markedly transcriptionally regulated by a protein (Pgp4) encoded by the *Chlamydia* virulence plasmid [65]. This plasmid is present in almost all *C. trachomatis* clinical isolates [66], and studies in animal models of infection showed that it is a virulence factor *in vivo*[67,68]. Additional studies are needed to understand if the putative effector function of CT142, CT143, and CT144 can partially explain the virulence role of the chlamydial plasmid. Furthermore, the predicted amino acid sequence of CT849 reveals a domain of unknown function (DUF720) that can only be found in *Chlamydia* proteins. In *C. trachomatis*, besides CT849, a DUF720 domain is found in CT847, a T3S effector that interacts with human *G*rap2 *c*yclin D-*i*nteracting *p*rotein (GCIP) [13], and in CT848, which has been indicated as a T3S substrate using *S. flexneri* as a heterologous system [21]. Therefore, this further supports a possible role of CT849 as an effector. In contrast with CT105, CT142, CT143, CT144 or CT849, no significant information is available or could be retrieved about CT053, CT338, CT429, or CT656.

CT161 is a possible T3S substrate and effector, but we could not detect significant levels of *ct161* mRNA during the developmental cycle of strain L2/434. The *ct161* gene is localized within the “plasticity zone”, a chromosomal region of rare high genetic diversity among *C. trachomatis* strains. In fact, although *C. trachomatis* includes strains showing remarkably different tropisms (strains that can spread into lymph nodes and cause lymphogranuloma venereum [LGV], such as L2/434, and strains causing infections usually restricted to the mucosa of the conjunctiva and genitals), their genomes are all highly similar [69]. Preliminary data indicate that, contrarily to what is seen in LGV strains, the *ct161* seems to be more expressed in some ocular and urogenital isolates (data not shown). We are currently investigating the possibility that *ct161* is a pseudogene in LGV strains, perhaps inactivated by a mutation in its promoter region. Interestingly, CT161 has been shown by yeast two-hybrid to bind CT274 (a possible chlamydial T3S chaperone) [70]. Another feature of this protein is that part of its amino acid sequence (residues 40–224, out of 246) shows 28% of identity to a region of Lda2/CT163 (residues 167–361, out of 548), a known *C. trachomatis* translocated protein [33].

Among the proteins for which we found a secretion signal but could not demonstrate their T3S as full-length proteins, we highlight CT153 and GrgA/CT504. Regarding CT153, this protein possesses a membrane attack complex/perforin (MACPF) domain [71], and there is previous evidence that it may be translocated by *C. trachomatis*[72], which is consistent with our data. The *ct504* gene has been recently shown to encode a transcriptional activator, GrgA [55]. Therefore, T3S of CT504_20_-TEM-1 could be false a positive. However, if GrgA is a T3S substrate, as our data suggests, it could have a function within the host cell or, more likely and similarly to what has been described in the T3SSs of *Yersinia*[73] or *Shigella*[74,75], it could be discarded by secretion once its intra-bacterial regulatory activity needs to be shut down.

We found T3S signals in 56% proteins analyzed (26 out of 46, including controls). This high percentage of proteins showing a T3S signal suggests that some should be false positives. It is conceivable that within a single bacterium non-secreted proteins possess T3S signals but are not targeted to the T3SS machinery because they also carry signals (e.g. DNA-, membrane-, or protein-binding) that preferentially direct them to other location within the bacterial cell. To help differentiating between true or false positives among chlamydial proteins carrying a T3S signal we analyzed their secretion as full-length proteins. This is because, as explained above in the Results section, not all proteins have folding characteristics compatible with T3S [59-62]. However, we cannot exclude that some of the *C. trachomatis* full-length proteins that were not type III secreted by *Yersinia* have a T3S chaperone that maintains them in a secretion-competent state [64] and enables their secretion during infection by *C. trachomatis*. Intriguingly, CT082 or CT694 have dedicated T3S chaperones, CT584 and Slc1, respectively [26], and, in agreement with what we previously observed [26], they were both secreted as full-length proteins in the absence of the chaperones. Considering that T3S chaperones have various functions [76,77], the chaperone role of CT584 or Slc1 should be different from maintaining their substrates in a secretion-competent state.

Eleven of the *Chlamydia* proteins that we analyzed have been previously studied for T3S using *S. flexneri* has a heterologous system [21]. In the majority of the cases the outcome of the experiments was identical; however, differently from what was shown in *Shigella*, we detected a T3S signal in the N-terminal of CT429 (which was also secreted as a full-length protein, and could be translocated into HeLa cells), GrgA/CT504, and CT779 and we did not detect a T3S signal in CT577. Evidence for a T3S signal in only one of the heterologous systems may suggest a false positive. However, there is a myriad of possible explanations for these discrepancies, when considering that different heterologous systems (*Shigella* and *Yersinia*) and reporter proteins (Cya and TEM-1) were used, and that the N-terminal regions in the hybrid proteins consisted in different lengths of amino acids and were in some cases from different *Chlamydia* species.

We compared the data from our T3S assays (including the controls, CT082, CT694, and RplJ) with predictions of T3S substrates by *in silico* methods (Effective T3S [28], SIEVE [29], Modlab [30], and T3_MM [56]) using resources available in the Web (Effective T3S, Modlab and T3_MM) and Table three in reference [29] (SIEVE), as detailed in Additional file 3: Table S3. When considering the analysis of T3S signals in TEM-1 hybrids, the vast majority of proteins (60%; 12 out of 20) in which we did not find a T3S signal were also predicted not to be secreted by each of the *in silico* methods. In contrast, the vast majority of proteins (58%; 15 out of 26) in which we detected a T3S signal were also predicted to be secreted by at least one of the *in silico* methods. The correlation between our experimental data and the *in silico* predictions was more striking when considering the T3S of full-length proteins. Among the 16 full-length proteins for which we could not find definitive evidence of T3S, 10 (i.e., 62.5%) were also predicted not to be secreted by each of the *in silico* methods, but among the 11 proteins that we showed or confirmed to be T3S substrates, 10 (i.e., 83%) were also predicted to be secreted by at least one of the *in silico* methods. Overall, this indicates some correlation between our experimental data and the *in silico* methods that predict T3S substrates. However, for many proteins, each of these *in silico* methods generates different predictions (see Additional file 3: Table S3). It is possible that the quantitative data on T3S such as the one we generated in this and in a previous study [45], can be used to normalize and improve the predictive value of such methods.

## Conclusions

We found 10 *C. trachomatis* proteins (CT053, CT105, CT142, CT143, CT144, CT161, CT338, CT429, CT656, and CT849) with a high likelihood of being T3S substrates, and therefore possible effectors delivered by the bacteria into host cells. For 6 of these proteins (CT053, CT105, CT142, CT143, CT338, and CT429), the hypothesis that they could be effectors was supported by their capacity of being translocated into host cells and by the expression of their encoding genes by *C. trachomatis*. The identification of all *C. trachomatis* effectors is a crucial step towards a comprehensive understanding of the mechanisms by which this pathogen subverts host cells. The recently developed methods for genetic manipulation of *Chlamydia* indicate that it should be possible to ectopically express candidate effectors in *C. trachomatis*[17,78], which would facilitate the analysis of their translocation into host cells. Our work highlights *C. trachomatis* proteins that should be prioritized in such studies, thus aiding the future identification of chlamydial effectors. Furthermore, the quantitative analysis of T3S of TEM-1 hybrid proteins that we carried out could help to further develop the *in silico* methods for identification of T3S substrates [28-30,56].

## Competing interests

The authors declare that they have no competing interests.

## Authors’ contributions

MdC, CM, FA, SVP, RM, and VB performed research and analyzed data. MdC, CM, FA, SVP, and RM performed T3S assays and VB carried out the RT-qPCR assays. MdC also performed the translocation assays and helped to write the paper. JPG and MJB designed research and analyzed data. LJM designed research, analyzed data and wrote the paper. All authors read and approved the final manuscript.

## Supplementary Material

Additional file 1: Table S1 Plasmids used and constructed in this work. Click here for file

Additional file 2: Table S2 Primers used in this work for construction of plasmids. Click here for file

Additional file 3: Table S3 Summary of results obtained in analyses of T3S signals in proteins of *Chlamydia trachomatis* and comparison to *in silico* prediction methods. Click here for file
