# Supplementary material for: Identification of type III secretion substrates of Chlamydia trachomatis using Yersinia enterocolitica as a heterologous system
Source: BMC Microbiol. 2014 Feb 17;14:40. doi: 10.1186/1471-2180-14-40 (PMC3931295; doi:10.1186/1471-2180-14-40)
Supplement: Additional file 1: Table S1 — Plasmids used and constructed in this work. [file 1471-2180-14-40-S1.pdf]

Table S1. Plasmids used and constructed in this work.

Plasmid	Description	Reference[a] or source
pCX340	Derivative of pBR322. Expresses mature TEM-1 β-lactamase	[1]
pLJM3	Low copy vector, derivative of pBBR1MCS-2 [2]. Expresses YopE under the control of its own promoter (P*yopE*).	[3]
pFA1	Derivative of pLJM3. Expresses IncA under the control of P*yopE*	[4]
pFA2	Derivative of pLJM3. Expresses IncC under the control of P*yopE*	[4]
pFA3	Derivative of pLJM3. Expresses TEM-1 under the control of P*yopE*	[4]
pFA8	Derivative of pLJM3. Expresses $IncA_{10}$-TEM-1 under the control of P*yopE*	This study
pFA9	Derivative of pLJM3. Expresses $IncA_{20}$-TEM-1 under the control of P*yopE*	[4]
pFA10	Derivative of pLJM3. Expresses $IncA_{40}$-TEM-1 under the control of P*yopE*	This study
pFA11	Derivative of pLJM3. Expresses $IncC_{10}$-TEM-1 under the control of P*yopE*	This study
pFA12	Derivative of pLJM3. Expresses $IncC_{20}$-TEM-1 under the control of P*yopE*	[4]
pFA13	Derivative of pLJM3. Expresses $IncC_{40}$-TEM-1 under the control of P*yopE*	This study
pFA14	Derivative of pLJM3. Expresses $YopE_{15}$-TEM-1 under the control of P*yopE*	This study
pFA19	Derivative of pLJM3. Expresses $SycT_{20}$-TEM-1 under the control of P*yopE*	[4]
pCM13	Derivative of pLJM3. Expresses $CT016_{20}$-TEM-1 under the control of P*yopE*	This study
pFA38	Derivative of pLJM3. Expresses $CT017_{20}$-TEM-1 under the control of P*yopE*	This study
pFA39	Derivative of pLJM3. Expresses $CT031_{20}$-TEM-1 under the control of P*yopE*	This study
pCM4	Derivative of pLJM3. Expresses $CT051_{20}$-TEM-1 under the control of P*yopE*	This study
pCM5	Derivative of pLJM3. Expresses $CT053_{20}$-TEM-1 under the control of P*yopE*	This study
pFA41	Derivative of pLJM3. Expresses $CT066_{20}$-TEM-1 under the control of P*yopE*	This study
pCM14	Derivative of pLJM3. Expresses $CT080_{20}$-TEM-1 under the control of P*yopE*	This study
pRM10	Derivative of pLJM3. Expresses $CT082_{20}$-TEM-1 under the control of P*yopE*	This study
pRM11	Derivative of pLJM3. Expresses $CT105_{20}$-TEM-1 under the control of P*yopE*	This study
pRM12	Derivative of pLJM3. Expresses $CT142_{20}$-TEM-1 under the control of P*yopE*	This study
pMC24	Derivative of pLJM3. Expresses $CT143_{20}$-TEM-1 under the control of P*yopE*	This study
pMC25	Derivative of pLJM3. Expresses $CT144_{20}$-TEM-1 under the control of P*yopE*	This study
pFA42	Derivative of pLJM3. Expresses $CT153_{20}$-TEM-1 under the control of P*yopE*	This study

Table S1. *Continued.*

Plasmid	Description	Reference or source
pCM6	Derivative of pLJM3. Expresses $CT161_{20}$-TEM-1 under the control of P*yopE*	This study
pCM15	Derivative of pLJM3. Expresses $CT172_{20}$-TEM-1 under the control of P*yopE*	This study
pCM16	Derivative of pLJM3. Expresses $CT203_{20}$-TEM-1 under the control of P*yopE*	This study
pFA43	Derivative of pLJM3. Expresses $CT273_{20}$-TEM-1 under the control of P*yopE*	This study
pCM17	Derivative of pLJM3. Expresses $CT277_{20}$-TEM-1 under the control of P*yopE*	This study
pCM18	Derivative of pLJM3. Expresses $CT289_{20}$-TEM-1 under the control of P*yopE*	This study
pRM13	Derivative of pLJM3. Expresses $CT309_{20}$-TEM-1 under the control of P*yopE*	This study
pCM19	Derivative of pLJM3. Expresses $CT330_{20}$-TEM-1 under the control of P*yopE*	This study
pCM20	Derivative of pLJM3. Expresses $CT338_{20}$-TEM-1 under the control of P*yopE*	This study
pFA44	Derivative of pLJM3. Expresses $CT386_{20}$-TEM-1 under the control of P*yopE*	This study
pFA45	Derivative of pLJM3. Expresses $CT425_{20}$-TEM-1 under the control of P*yopE*	This study
pFA46	Derivative of pLJM3. Expresses $CT429_{20}$-TEM-1 under the control of P*yopE*	This study
pFA47	Derivative of pLJM3. Expresses $CT504_{20}$-TEM-1 under the control of P*yopE*	This study
pFA48	Derivative of pLJM3. Expresses $CT538_{20}$-TEM-1 under the control of P*yopE*	This study
pCM21	Derivative of pLJM3. Expresses $CT568_{20}$-TEM-1 under the control of P*yopE*	This study
pFA49	Derivative of pLJM3. Expresses $CT577_{20}$-TEM-1 under the control of P*yopE*	This study
pRM16	Derivative of pLJM3. Expresses $CT583_{20}$-TEM-1 under the control of P*yopE*	This study
pFA50	Derivative of pLJM3. Expresses $CT584_{20}$-TEM-1 under the control of P*yopE*	This study
pFA51	Derivative of pLJM3. Expresses $CT590_{20}$-TEM-1 under the control of P*yopE*	This study
pFA52	Derivative of pLJM3. Expresses $CT631_{20}$-TEM-1 under the control of P*yopE*	This study
pFA53	Derivative of pLJM3. Expresses $CT635_{20}$-TEM-1 under the control of P*yopE*	This study
pCM8	Derivative of pLJM3. Expresses $CT656_{20}$-TEM-1 under the control of P*yopE*	This study
pRM20	Derivative of pLJM3. Expresses $CT696_{20}$-TEM-1 under the control of P*yopE*	This study
pCM9	Derivative of pLJM3. Expresses $CT702_{20}$-TEM-1 under the control of P*yopE*	This study
pFA57	Derivative of pLJM3. Expresses $CT768_{20}$-TEM-1 under the control of P*yopE*	This study

Table S1. *Continued.*

Plasmid	Description	Reference or source
pCM22	Derivative of pLJM3. Expresses $CT779_{20}$-TEM-1 under the control of P*yopE*	This study
pFA58	Derivative of pLJM3. Expresses $CT814_{20}$-TEM-1 under the control of P*yopE*	This study
pFA59	Derivative of pLJM3. Expresses $CT837_{20}$-TEM-1 under the control of P*yopE*	This study
pCM11	Derivative of pLJM3. Expresses $CT845_{20}$-TEM-1 under the control of P*yopE*	This study
pFA60	Derivative of pLJM3. Expresses $CT849_{20}$-TEM-1 under the control of P*yopE*	This study
pRM15	Derivative of pLJM3. Expresses $CT863_{20}$-TEM-1 under the control of P*yopE*	This study
pFA37	Derivative of pLJM3. Expresses $RplJ_{20}$-TEM-1 under the control of P*yopE*	This study
pFA55	Derivative of pLJM3. Expresses $CT694_{20}$-TEM-1 under the control of P*yopE*	This study
pMC1	Derivative of pLJM3. Expresses CT016-HA under the control of P*yopE*	This study
pMC7	Derivative of pLJM3. Expresses CT051-HA under the control of P*yopE*	This study
pMC2	Derivative of pLJM3. Expresses CT053-HA under the control of P*yopE*	This study
pMC3	Derivative of pLJM3. Expresses CT080-HA under the control of P*yopE*	This study
pRM1	Derivative of pLJM3. Expresses CT082-HA under the control of P*yopE*	This study
pRM7	Derivative of pLJM3. Expresses CT105-HA under the control of P*yopE*	This study
pRM2	Derivative of pLJM3. Expresses CT142-HA under the control of P*yopE*	This study
pMC13	Derivative of pLJM3. Expresses CT143-HA under the control of P*yopE*	This study
pMC5	Derivative of pLJM3. Expresses CT144-HA under the control of P*yopE*	This study
pFA62	Derivative of pLJM3. Expresses CT153-HA under the control of P*yopE*	This study
pMC6	Derivative of pLJM3. Expresses CT161-HA under the control of P*yopE*	This study
pMC14	Derivative of pLJM3. Expresses CT172-HA under the control of P*yopE*	This study
pMC20	Derivative of pLJM3. Expresses CT203-HA under the control of P*yopE*	This study
pFA63	Derivative of pLJM3. Expresses CT273-HA under the control of P*yopE*	This study
pMC22	Derivative of pLJM3. Expresses CT277-HA under the control of P*yopE*	This study
pMC18	Derivative of pLJM3. Expresses CT289-HA under the control of P*yopE*	This study
pRM3	Derivative of pLJM3. Expresses CT309-HA under the control of P*yopE*	This study

Table S1. *Continued.*

Plasmid	Description	Reference or source
pMC15	Derivative of pLJM3. Expresses CT330-HA under the control of P*yopE*	This study
pMC19	Derivative of pLJM3. Expresses CT338-HA under the control of P*yopE*	This study
pFA64	Derivative of pLJM3. Expresses CT386-HA under the control of P*yopE*	This study
pFA65	Derivative of pLJM3. Expresses CT425-HA under the control of P*yopE*	This study
pFA66	Derivative of pLJM3. Expresses CT429-HA under the control of P*yopE*	This study
pFA67	Derivative of pLJM3. Expresses CT504-HA under the control of P*yopE*	This study
pMC7	Derivative of pLJM3. Expresses CT568-HA under the control of P*yopE*	This study
pRM6	Derivative of pLJM3. Expresses CT583-HA under the control of P*yopE*	This study
pFA68	Derivative of pLJM3. Expresses CT631-HA under the control of P*yopE*	This study
pMC8	Derivative of pLJM3. Expresses CT656-HA under the control of P*yopE*	This study
pCM12	Derivative of pLJM3. Expresses CT694-HA under the control of P*yopE*	This study
pRM18	Derivative of pLJM3. Expresses CT696-HA under the control of P*yopE*	This study
pMC9	Derivative of pLJM3. Expresses CT702-HA under the control of P*yopE*	This study
pMC10	Derivative of pLJM3. Expresses CT779-HA under the control of P*yopE*	This study
pMC21	Derivative of pLJM3. Expresses CT845-HA under the control of P*yopE*	This study
pFA70	Derivative of pLJM3. Expresses CT849-HA under the control of P*yopE*	This study
pRM5	Derivative of pLJM3. Expresses CT863-HA under the control of P*yopE*	This study
pFA61	Derivative of pLJM3. Expresses RplJ-HA under the control of P*yopE*	This study

1. Charpentier X, Oswald E: **Identification of the secretion and translocation domain of the enteropathogenic and enterohemorrhagic *Escherichia coli* effector Cif, using TEM-1 beta-lactamase as a new fluorescence-based reporter**. *J Bacteriol* 2004, **186**(16):5486-5495.
2. Kovach ME, Elzer PE, Hill DS, Robertson GT, Farris MA, Roop II RM, Peterson KM: **Four new derivatives of the broad-host-range cloning vector pBBR1MCS, carrying different antibiotic-resistance cassettes**. *Gene* 1995, **166**:175-176.
3. Marenne MN, Journet L, Mota LJ, Cornelis GR: **Genetic analysis of the formation of the Ysc-Yop translocation pore in macrophages by *Yersinia enterocolitica*: role of LcrV, YscF and YopN**. *Microb Pathog* 2003, **35**(6):243-258.
4. Almeida F, Borges V, Ferreira R, Borrego MJ, Gomes JP, Mota LJ: **Polymorphisms in Inc Proteins and Differential Expression of *inc* Genes among *Chlamydia trachomatis* Strains Correlate with Invasiveness and Tropism of Lymphogranuloma Venereum Isolates**. *J Bacteriol* 2012, **194**(23):6574-6585.
