# Supplementary material for: Identification of type III secretion substrates of Chlamydia trachomatis using Yersinia enterocolitica as a heterologous system
Source: BMC Microbiol. 2014 Feb 17;14:40. doi: 10.1186/1471-2180-14-40 (PMC3931295; doi:10.1186/1471-2180-14-40)
Supplement: Additional file 2: Table S2 — Primers used in this work for construction of plasmids. [file 1471-2180-14-40-S2.pdf]

Table S2. Primers used in this work for construction of plasmids.

Code	Description	[a]Sequence (5’-3’)	Restriction enzyme
28	Used to amplify the 5’ region of genes cloned in pLJM3 to construct TEM-1 hybrids by overlapping PCR	GATTAAGTTGGGTAACGCC	-
30	Used to construct pFA8 by overlapping PCR	CACCAGCGTTTCTGGGTGAGGAGTCACGATTAGAGTAGG	-
31	Used to construct pFA8 by overlapping PCR	CCTACTCTAATCGTGACTCCTCACCCAGAAACGCTGGTG	-
34	Used to construct pFA8 by overlapping PCR	CACCAGCGTTTCTGGGTGGGAGGCAATGGCTGCTATTTTC	-
35	Used to construct pFA10 by overlapping PCR	GAAAATAGCAGCCATTGCCTCCCACCCAGAAACGCTGGTG	-
36	Used to construct pFA11 by overlapping PCR	CACCAGCGTTTCTGGGTGGTGTGCTATATCGGACATAGAGTACG	-
37	Used to construct pFA11 by overlapping PCR	CGTACTCTATGTCCGATATAGCACACCACCCAGAAACGCTGGTG	-
40	Used to construct pFA13 by overlapping PCR	CACCAGCGTTTCTGGGTGTAAAGAGCCCACGGCAGAAGG	-
41	Used to construct pFA13 by overlapping PCR	CCTTCTGCCGTGGGCTCTTTACACCCAGAAACGCTGGTG	-
42	Used to construct pFA14 by overlapping PCR	CACCAGCGTTTCTGGGTGTGCCGGCAGGGGCAGTGATGTAG	-
43	Used to construct pFA14 by overlapping PCR	CTACATCACTGCCCCTGCCGGCACACCCAGAAACGCTGGTG	-
60	Used to amplify *tem-1* to construct TEM-1 hybrids	CCCAAGCTTTTACCAATGCTTAATCAGTGAGG	HindIII
67	Used to amplify *tem-1* to construct TEM-1 hybrids	GATCCTCGAGTTACCAATGCTTAATCAGTGAGG	XhoI
103	Used to construct pFA38	GGAATTCCATATGCTCATTTTTGCCCTTTCTTGTGGGGCAGATGCCTGCTTATGTGCTGCGGATCTTTCCCACCCAGAAACGCTGGTG	NdeI
104	Used to construct pFA39	GGAATTCCATATGGCTAGAAAAGATCGTTTAACTAATGAAAGACTGAATAAGCTATTTGATAGCCCCTTTCACCCAGAAACGCTGGTG	NdeI
106	Used to construct pFA41	GGAATTCCATATGGCTACGGCACAGATTACTATCCAAGAAGAAATAGAGCAGCTCATAACTAAAGCGATTCACCCAGAAACGCTGGTG	NdeI
107	Used to construct pFA42	GGAATTCCATATGACTAAGCCTTCTTTCTTATACGTTATTCAACCTTTTTCCGTATTTAATCCACGATTACACCCAGAAACGCTGGTG	NdeI
108	Used to construct pFA43	GGAATTCCATATGGTCGAAATTTTTAACTATAGTACCTCGGTATACGAGAAACACAGCTCTACGAACAAGCACCCAGAAACGCTGGTG	NdeI

Table S2. ***Continued.***

Code	Description	[a]Sequence (5’-3’)	Restriction enzyme
109	Used to construct pFA44	GGAATTCCATATGCAAATTCCAAGAAGTGTTGGCACACATGATGGTTCTTTTCACGCAGATGAAGTGACGCACCCAGAAACGCTGGTG	NdeI
110	Used to construct pFA45	GGAATTCCATATGCGCAGATCTGTTTGTTACGTTACTCCTTCAGTTGCTAGGGCTGGTCAAATTTCTACCCACCCAGAAACGCTGGTG	NdeI
111	Used to construct pFA46	GGAATTCCATATGACGACATATCCTGTACCTCAAAATCCTCTTTTATTACGCGTTCTACGCCTGATGGACCACCCAGAAACGCTGGTG	NdeI
112	Used to construct pFA47	GGAATTCCATATGTATTTTACAAGAGATCCAGTCATAGAGACTGTTATTACATCTAGAGAAGGATATAAGCACCCAGAAACGCTGGTG	NdeI
113	Used to construct pFA48	GGAATTCCATATGAATATTTCTGGAAGTATCAAACAAAAACTTCTCCAGTTTTTGAAAAAGCAAAAATCCCACCCAGAAACGCTGGTG	NdeI
114	Used to construct pFA49	GGAATTCCATATGAGTAAAAAACATAAGCACAAGCAAGCACACACTTCTTCCAAACCCAAAGTAGAACCTCACCCAGAAACGCTGGTG	NdeI
115	Used to construct pFA50	GGAATTCCATATGACGACGAAACCCAAAACTCTAGAAATCGATAACAACACGTTCCTGCTTTTGGAAGGCCACCCAGAAACGCTGGTG	NdeI
116	Used to construct pFA51	GGAATTCCATATGTCTCGTTTGGATGTTTCTGTATTTGATTCCTTAGCTAATAAAGAAAAAGCTTCCTTACACCCAGAAACGCTGGTG	NdeI
117	Used to construct pFA52	GGAATTCCATATGAAAACGTTAATTGATAACAACATCGTCAGATTCAAAAATATTTCTAAAACCAAACAGCACCCAGAAACGCTGGTG	NdeI
118	Used to construct pFA53	GGAATTCCATATGAAAAATAATTCCGCTCAAAAAATTATAGATTCTATAAAACAAATTCTCTCTATTTATCACCCAGAAACGCTGGTG	NdeI
102	Used to construct pFA37	GGAATTCCATATGAAAGAAGAGAAAAAGTTGCTGCTTCGCGAGGTTGAAGAAAAGATAACCGCTTCTCAACACCCAGAAACGCTGGTG	NdeI
120	Used to construct pFA55	GGAATTCCATATGAGTATTCGACCTACTAATGGGAGTGGAAATGGATACCCGTCTATTAATCCTTCTAACCACCCAGAAACGCTGGTG	NdeI
122	Used to construct pFA57	GGAATTCCATATGGATATTCCAGAACAGGGCTCAAATACACCAGAAGTAGAGCAAGCAGCTTGCTGCAATCACCCAGAAACGCTGGTG	NdeI

Table S2. ***Continued.***

Code	Description	[a]Sequence (5'-3')	Restriction enzyme
123	Used to construct pFA58	GGAATTCCATATGTTCAGAAGCCAAAAACCTAAAAAAAATAAATGTTGCTTATGGTTGCGAGGCGTCCTACACCCAGAAACGCTGGTG	NdeI
124	Used to construct pFA59	GGAATTCCATATGGAAAAGACTCGTAAGTTTGAAAAAGCTTTAGAGAATTTAGAGCAGTTAAAAAAGATTCACCCAGAAACGCTGGTG	NdeI
125	Used to construct pFA60	GGAATTCCATATGTCAGCACCAACCTCACAGGTAGGAGACACACAATACGTCTCCTCGCTACCTCCTTTACACCCAGAAACGCTGGTG	NdeI
169	Used to construct pRM1	GGAATTCCATATGTCAATTTCTGGAAGTGG	NdeI
170	Used to construct pRM1	GATCCTCGAGTCAAGCATAATCAGGAACATCATACGGATATGAATCGCCGCCTGCATCCTC	XhoI
171	Used to construct pRM10 by overlapping PCR	CACCAGCGTTTCTGGGTGGATGGATGGGTCAAAATCAGG	-
172	Used to construct pRM10 by overlapping PCR	CCTGATTTTGACCCATCCATCCACCCAGAAACGCTGGTG	-
173	Used to construct pRM7	GGAATTCCATATGTCATTTGGTATTGGTAG	NdeI
174	Used to construct pRM7	GATCGGTACCCTAAGCATAATCAGGAACATCATACGGATAGATAGAGGAGCTTTGCACACC	KpnI
175	Used to construct pRM11 by overlapping PCR	GCCGTTTGTGTGGTTCATCACACCCAGAAACGCTGGTG	-
176	Used to construct pRM11 by overlapping PCR	CACCAGCGTTTCTGGGTGTGATGAACCACACAAACGGC	-
177	Used to construct pRM2	GGAATTCCATATGAGTGATTCTGACAAAATTATTAATG	NdeI
178	Used to construct pRM2	GATCGGTACCTTAAGCATAATCAGGAACATCATACGGATATCCTCCTATCTCTGGGTATACG	KpnI
179	Used to construct pRM12 by overlapping PCR	CGACTTTAATACAACTGTTCATCACCCAGAAACGCTGGTG	-
180	Used to construct pRM12 by overlapping PCR	CACCAGCGTTTCTGGGTGATGAACAGTTGTATTAAAGTCG	-
181	Used to construct pRM3	GGAATTCCATATGAATCAGTATTATTTTTTATCC	NdeI
182	Used to construct pRM3	GATCAAGCTTCTAAGCATAATCAGGAACATCATACGGATACCATCTGATTCCTTTCTCC	HindIII
183	Used to construct pRM13 by overlapping PCR	CAACCCGAATCTTCTCCTCACCCAGAAACGCTGGTG	-
184	Used to construct pRM13 by overlapping PCR	CACCAGCGTTTCTGGGTGAGGAGAAGATTCGGGTTG	-
185	Used to construct pRM18	GGAATTCCATATGCTATTAGATTCTCGTTTCCC	NdeI

Table S2. *Continued.*

Code	Description	[a]Sequence (5'-3')	Restriction enzyme
186	Used to construct pRM18	GATCCTCGAGCTAAGCATAATCAGGAACATCATACGGATAACGAGCTTCCTTACGGAAAGTTCC	XhoI
187	Used to construct pRM20 by overlapping PCR	CGTATCCTAGAATTAGTCATCCACCCAGAAACGCTGGTG	-
188	Used to construct pRM20 by overlapping PCR	CACCAGCGTTTCTGGGTGGATGACTAATTCTAGGATACG	-
193	Used to construct pRM5	GGAATTCCATATGGATACTCCCACACCCC	NdeI
194	Used to construct pRM5	GATCCTCGAGTTAAGCATAATCAGGAACATCATACGGATAGGGACGCATGTTGTAG	XhoI
195	Used to construct pRM15 by overlapping PCR	GCTTCTCTTAAGGGAGAACCACACCCAGAAACGCTGGTG	-
196	Used to construct pRM15 by overlapping PCR	CACCAGCGTTTCTGGGTGTGGTTCTCCCTTAAGAGAAGC	-
197	Use to construct pRM6	GGAATTCCATATGGGAAATATTAAAACCC	NdeI
198	Use to construct pRM6	GATCAAGCTTTTAAGCATAATCAGGAACATCATACGGATATCGATTTCTAGAGTTTTGGG	HindIII
199	Used to construct pRM16 by overlapping PCR	AAACCTACACCCGATAAAATGCACCCAGAAACGCTGGTG	-
200	Used to construct pRM16 by overlapping PCR	CACCAGCGTTTCTGGGTGCATTTTATCGGGTGTAGGTTT	-
206	Used to construct pFA61	GAATTCCATATGAAAGAAGAGAAAAAGTTGC	NdeI
207	Used to construct pFA61	GATCCTCGAGTTAAGCATAATCAGGAACATCATACGGATACTCTTGAGTTTTTCTGC	XhoI
208	Used to construct pFA62	GAATTCCATATGACTAAGCCTTCTTTCTTATACG	NdeI
209	Used to construct pFA62	GATCCTCGAGTTAAGCATAATCAGGAACATCATACGGATAATAACCTGAAGATTTTTTAA	XhoI
212	Used to construct pFA63	GAATTCCATATGGTCGAAATTTTTAACTATAGTACC	NdeI
213	Used to construct pFA63	GATCCTCGAGTTAAGCATAATCAGGAACATCATACGGATAACCTTGTACGAATTGAAAAATACG	XhoI
214	Used to construct pFA64	GAATTCCATATGCAAATTCCAAGAAGTGTTGGC	NdeI
215	Used to construct pFA64	CCCAAGCTTAAGCATAATCAGGAACATCATACGGATATACTAATCTCTGCTGTTTTAAC	HindIII
216	Used to construct pFA65	GAATTCCATATGCGCAGATCTGTTTGTTACG	NdeI
217	Used to construct pFA65	CCCAAGCTTAAGCATAATCAGGAACATCATACGGATAGTTAAGATCTATCCAAATAGG	HindIII

Table S2. ***Continued.***

Code	Description	[a]Sequence (5'-3')	Restriction enzyme
219	Used to construct pFA66	GAATTCCATATGACGACATATCCTGTACC	NdeI
220	Used to construct pFA66	GATCCTCGAGTTAAGCATAATCAGGAACATCATACGGATATGAACGGCTCTTCTTACG	XhoI
221	Used to construct pFA67	GAATTCCATATGTATTTTACAAGAGATCC	NdeI
222	Used to construct pFA67	GATCCTCGAGTTAAGCATAATCAGGAACATCATACGGATACTCTTCTGAAGAAATACTGTC	XhoI
223	Used to construct pFA68	GAATTCCATATGAAAACGTTAATTGATAAC	NdeI
224	Used to construct pFA68	CCCAAGCTTAAGCATAATCAGGAACATCATACGGATATAAACAAATAATTCCTTCAAACTG	HindIII
227	Used to construct pFA70	GAATTCCATATGTCAGCACCAACCTCACAGG	NdeI
228	Used to construct pFA70	CCCAAGCTTAAGCATAATCAGGAACATCATACGGATAAGACAGGGGTTTATTTAATTGG	HindIII
229	Used to construct pMC1	GGAATTCCATATGAAAGTCAAAATTAATGATC	NdeI
230	Used to construct pMC1	GATCAAGCTTTTAAGCATAATCAGGAACATCATACGGATAAGTATAAAGAACAGCTTTCACG	HindIII
233	Used to construct pMC7	GGAATTCCATATGAATAAAAAAGAACGAATTAATAAAAAAAACGC	NdeI
234	Used to construct pMC7	GATCGGTACCCTAAGCATAATCAGGAACATCATACGGATATACTATCTTATTTTTAATC	KpnI
235	Used to construct pMC2	GGAATTCCATATGAAAAGTGAGCGTTTAAAAAAATTAGAATCAGAGC	NdeI
236	Used to construct pMC2	GATCAAGCTTTTAAGCATAATCAGGAACATCATACGGATACCATTCATTCGCGTCAGG	HindIII
237	Used to construct pMC3	GGAATTCCATATGAAAAAAAGAAGCAGTCGC	NdeI
238	Used to construct pMC3	GATCAAGCTTCTAAGCATAATCAGGAACATCATACGGATACAGTTTCACAGAATATCGCC	HindIII
241	Used to construct pMC13	GGAATTCCATATGAAGAAACCAGTATTTACAGG	NdeI
242	Used to construct pMC13	GATCCTCGAGTTAAGCATAATCAGGAACATCATACGGATAATCTGCCTCCTTATAAGAAG	XhoI
243	Used to construct pMC5	GGAATTCCATATGACAACACCAGATAATAATAC	NdeI
244	Used to construct pMC5	GATCAAGCTTTTAAGCATAATCAGGAACATCATACGGATAAGGAACAACAGGTAGCCG	HindIII

Table S2. *Continued.*

Code	Description	[a]Sequence (5’-3’)	Restriction enzyme
245	Used to construct pMC6	GGAATTCCATATGGCTAGAAAACCTTTAGTAG	NdeI
246	Used to construct pMC6	GATCAAGCTTTCAAGCATAATCAGGAACATCATACGGATAGTCATAAAAATTTTCCATTTCTG	HindIII
247	Used to construct pMC14	GGAATTCCATATGTTTTTCTTGGCAAAAAGAC	NdeI
248	Used to construct pMC14	GATCCTCGAGTCAAGCATAATCAGGAACATCATACGGATACTCTTGATAGTCTTGCATGG	XhoI
249	Used to construct pMC20	GGAATTCCATATGGAAATCTCCCATATTTTGG	NdeI
250	Used to construct pMC20	GATCCTCGAGCTAAGCATAATCAGGAACATCATACGGATATGACTCAAAAGGAAATC	XhoI
251	Used to construct pMC22	GGAATTCCATATGTCTGAGAGAAAGGTTGAG	NdeI
252	Used to construct pMC22	GATCCTCGAGCTAAGCATAATCAGGAACATCATACGGATAATGAGCAAGAGGAAATAGAC	XhoI
253	Used to construct pMC18	GGAATTCCATATGGTGATCCCTAAGGTGG	NdeI
254	Used to construct pMC18	GATCGGTACCTTAAGCATAATCAGGAACATCATACGGATATGGTTGCGTTGAGCCTCC	KpnI
255	Used to construct pMC15	GGAATTCCATATGCAGGAAATCTCGGTACC	NdeI
256	Used to construct pMC15	GATCCTCGAGTCAAGCATAATCAGGAACATCATACGGATATACAGATTCCCCAGGGATAAAAGG	XhoI
257	Used to construct pMC19	GGAATTCCATATGGTGTATAGTTATAAAGG	NdeI
258	Used to construct pMC19	GATCGGTACCCTAAGCATAATCAGGAACATCATACGGATAAGGTTTTTGAGATAAAAGATACTG	KpnI
261	Used to construct pMC7	GGAATTCCATATGAACAGCGAGGGTAAGG	NdeI
262	Used to construct pMC7	GATCAAGCTTTTAAGCATAATCAGGAACATCATACGGATATCTTTCTAAACATAAACACC	HindIII
263	Used to construct pMC8	GGAATTCCATATGGACACGCAATTCATAGC	NdeI
264	Used to construct pMC8	GATCAAGCTTTCAAGCATAATCAGGAACATCATACGGATAATCTCTGTATACCGAACGC	HindIII
265	Used to construct pMC9	GGAATTCCATATGCACTACGAACCCTATGATG	NdeI
266	Used to construct pMC9	GATCAAGCTTTTAAGCATAATCAGGAACATCATACGGATATATGAAAGTAGACCATTTAGACC	HindIII

Table S2. ***Continued.***

Code	Description	aSequence (5’-3’)	Restriction enzyme
267	Used to construct pMC10	GGAATTCCATATGCATTCACTTACTGTTTTTCAAG	NdeI
268	Used to construct pMC10	GATCAAGCTTCTAAGCATAATCAGGAACATCATACGGATATGACTTCTGCATAGAGGC	HindIII
277	Used to construct pMC21	GGAATTCCATATGTCGCTAGGGACGACGATTG	NdeI
278	Used to construct pMC21	GATCAAGCTTCTAAGCATAATCAGGAACATCATACGGATAATCCTTACAGAGGCTCG	HindIII
325	Used to construct pMC24 by overlapping PCR	GGGATAAGTACAGAAGAAGGACACCCAGAAACGCTGGTG	-
326	Used to construct pMC24 by overlapping PCR	CACCAGCGTTTCTGGGTGTCCTTCTTCTGTACTTATCCC	-
327	Used to construct pMC25 by overlapping PCR	CCGACATTTGTACGTTTGAATCACCCAGAAACGCTGGTG	-
328	Used to construct pMC25 by overlapping PCR	CACCAGCGTTTCTGGGTGATTCAAACGTACAAATGTCGG	-
690	Used to construct pCM12	GAATTCCATATGAGTATTCGACCTACTAATGG	NdeI
691	Used to construct pCM12	CCCAAGCTTAAGCATAATCAGGAACATCATACGGATAGTCTAAGAAAACAGAAGAAG	HindIII
696	Used to construct pCM13 by overlapping PCR	CACCAGCGTTTCTGGGTGCCATCGAGCAGAAATGTATGG	-
697	Used to construct pCM13 by overlapping PCR	CCATACATTTCTGCTCGATGGCACCCAGAAACGCTGGTG	-
700	Used to construct pCM4 by overlapping PCR	CACCAGCGTTTCTGGGTGTGTATTTCGTTGGATTTTCG	-
701	Used to construct pCM4 by overlapping PCR	CGAAAATCCAACGAAATACACACCCAGAAACGCTGGTG	-
702	Used to construct pCM5 by overlapping PCR	CACCAGCGTTTCTGGGTGCATCCACTGGGTAAGATC	-
703	Used to construct pCM5 by overlapping PCR	GATCTTACCCAGTGGATGCACCCAGAAACGCTGGTG	-
704	Used to construct pCM14 by overlapping PCR	CACCAGCGTTTCTGGGTGATAGTTTCCCGTCTTACG	-
705	Used to construct pCM14 by overlapping PCR	CGTAAGACGGGAAACTATCACCCAGAAACGCTGGTG	-
706	Used to construct pCM6 by overlapping PCR	CACCAGCGTTTCTGGGTGATCACAAATAGTCCGATACTTGGG	-
707	Used to construct pCM6 by overlapping PCR	CCCAAGTATCGGACTATTTGTGATCACCCAGAAACGCTGGTG	-
708	Used to construct pCM15 by overlapping PCR	CACCAGCGTTTCTGGGTGCTTTTTGAGTAAGAAAGG	-
709	Used to construct pCM15 by overlapping PCR	CCTTTCTTACTCAAAAAGCACCCAGAAACGCTGGTG	-
710	Used to construct pCM16 by overlapping PCR	CACCAGCGTTTCTGGGTGTTCTCTAGGAAGAACCCC	-
711	Used to construct pCM16 by overlapping PCR	GGGGTTCTTCCTAGAGAACACCCAGAAACGCTGGTG	-

Table S2. *Continued.*

Code	Description	[a]Sequence (5'-3')	Restriction enzyme
712	Used to construct pCM17 by overlapping PCR	CACCAGCGTTTCTGGGTGAAATTTTAA TAATTTGTTACG	-
713	Used to construct pCM17 by overlapping PCR	CGTAACAAATTATTAAAATTTCACCCA GAAACGCTGGTG	-
714	Used to construct pCM18 by overlapping PCR	CACCAGCGTTTCTGGGTGAGTAAGCTT GTAACCCAG	-
715	Used to construct pCM18 by overlapping PCR	CTGGGTTACAAGCTTACTCACCCAGAA ACGCTGGTG	-
716	Used to construct pCM19 by overlapping PCR	CACCAGCGTTTCTGGGTGCAGAAGGCG CTGTATAAC	-
717	Used to construct pCM19 by overlapping PCR	GTTATACAGCGCCTTCTGCACCCAGAA ACGCTGGTG	-
718	Used to construct pCM20 by overlapping PCR	CACCAGCGTTTCTGGGTGTCCTACAGT TTCAAAAGG	-
719	Used to construct pCM20 by overlapping PCR	CCTTTTGAAACTGTAGGACACCCAGAA ACGCTGGTG	-
722	Used to construct pCM21 by overlapping PCR	CACCAGCGTTTCTGGGTGTAGTAAAAA ACAACGTTTTCG	-
723	Used to construct pCM21 by overlapping PCR	CGAAAACGTTGTTTTTTACTACACCCA GAAACGCTGGTG	-
724	Used to construct pCM8 by overlapping PCR	CACCAGCGTTTCTGGGTGTGAAACAGA AGTAACTTC	-
725	Used to construct pCM8 by overlapping PCR	GAAGTTACTTCTGTTTCACACCCAGAA ACGCTGGTG	-
726	Used to construct pCM9 by overlapping PCR	CACCAGCGTTTCTGGGTGGCAGATGAG ATGATCTAG	-
727	Used to construct pCM9 by overlapping PCR	CTAGATCATCTCATCTGCCACCCAGAA ACGCTGGTG	-
728	Used to construct pCM22 by overlapping PCR	CACCAGCGTTTCTGGGTGGGGGTAGGG CTTTTCTGTGTAGCG	-
729	Used to construct pCM22 by overlapping PCR	CGCTACACAGAAAAGCCCTACCCCCAC CCAGAAACGCTGGTG	-
732	Used to construct pCM11 by overlapping PCR	CACCAGCGTTTCTGGGTGCATAGGCTG TAAAAGATC	-
733	Used to construct pCM11 by overlapping PCR	GATCTTTTACAGCCTATGCACCCAGAA ACGCTGGTG	-

[a]Sites of restriction enzymes are underlined
